# 1-Benzoyl-3-methyl-3-pentyl­thio­urea

**DOI:** 10.1107/S1600536811013365

**Published:** 2011-04-16

**Authors:** N. Gunasekaran, P. Jerome, R. Karvembu, Seik Weng Ng, Edward R. T. Tiekink

**Affiliations:** a Department of Chemistry, National Institute of Technology, Tiruchirappalli 620 015, India; b Department of Chemistry, University of Malaya, 50603 Kuala Lumpur, Malaysia

## Abstract

Two independent mol­ecules comprise the asymmetric unit of the title compound, C_14_H_20_N_2_OS. These differ in the relative orientations of the pentyl chains [C—C—C—C torsion angles = −176.7 (3) and 176.4 (3)°]. Significant twists are evident in each mol­ecule, the dihedral angles formed between the thio­urea and amide residues being 53.47 (17) and 55.81 (17)°. In the crystal, each mol­ecule self-associates *via* a centrosymmetric eight-membered {⋯HNC=S}_2_ synthon, and these are connected into a supra­molecular chain along [110] *via* C—H⋯O contacts. Disorder is noted for one of the independent mol­ecules in that two orientations (50:50) were resolved for its benzene ring.

## Related literature

For the coordination potental of thio­urea derivatives, see: Pisiewicz *et al.* (2010 ▶). For pharmaceutical applications of thio­ruea deriavives, see: Venkatachalam *et al.* (2004 ▶); Bruce *et al.* (2007 ▶). For applications of thio­urea derivatives in catalysis, see: Gunasekaran *et al.* (2010 ▶, 2011 ▶). For closely related structures, see: Gunasekaran *et al.* (2010*a* ▶,*b* ▶,*c* ▶).
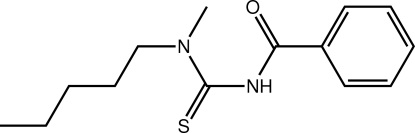


## Experimental

### 

#### Crystal data

C_14_H_20_N_2_OS
*M* *_r_* = 264.38
Triclinic, 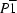

*a* = 9.0992 (6) Å
*b* = 10.5297 (6) Å
*c* = 16.4038 (8) Å
α = 75.784 (5)°
β = 77.831 (5)°
γ = 82.877 (5)°
*V* = 1484.98 (15) Å^3^
*Z* = 4
Mo *K*α radiation
μ = 0.21 mm^−1^
*T* = 295 K
0.25 × 0.20 × 0.15 mm

#### Data collection

Agilent Supernova Dual diffractometer with an Atlas detector
Absorption correction: multi-scan (*CrysAlis PRO*; Agilent, 2010 ▶) *T*_min_ = 0.853, *T*_max_ = 1.000
11884 measured reflections
6585 independent reflections
3555 reflections with *I* > 2σ(*I*)
*R*_int_ = 0.034

#### Refinement

*R*[*F* ^2^ > 2σ(*F* ^2^)] = 0.062
*wR*(*F* ^2^) = 0.184
*S* = 1.03
6585 reflections
321 parameters
37 restraints
H-atom parameters constrained
Δρ_max_ = 0.33 e Å^−3^
Δρ_min_ = −0.27 e Å^−3^

### 

Data collection: *CrysAlis PRO* (Agilent, 2010 ▶); cell refinement: *CrysAlis PRO*; data reduction: *CrysAlis PRO*; program(s) used to solve structure: *SHELXS97* (Sheldrick, 2008 ▶); program(s) used to refine structure: *SHELXL97* (Sheldrick, 2008 ▶); molecular graphics: *ORTEP-3* (Farrugia, 1997 ▶), *DIAMOND* (Brandenburg, 2006 ▶) and *QMOL* (Gans & Shalloway, 2001 ▶); software used to prepare material for publication: *publCIF* (Westrip, 2010 ▶).

## Supplementary Material

Crystal structure: contains datablocks global, I. DOI: 10.1107/S1600536811013365/hb5837sup1.cif

Structure factors: contains datablocks I. DOI: 10.1107/S1600536811013365/hb5837Isup2.hkl

Additional supplementary materials:  crystallographic information; 3D view; checkCIF report

## Figures and Tables

**Table 1:** Hydrogen-bond geometry (Å, °)

*D*—H⋯*A*	*D*—H	H⋯*A*	*D*⋯*A*	*D*—H⋯*A*
N1—H1⋯S1^i^	0.88	2.87	3.604 (2)	143
N3—H3⋯S2^ii^	0.88	2.72	3.449 (2)	141
C10—H10b⋯O2^iii^	0.97	2.55	3.397 (4)	146

Symmetry codes: (i) 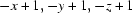; (ii) 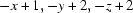; (iii) 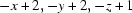.

**Fig. 1.**
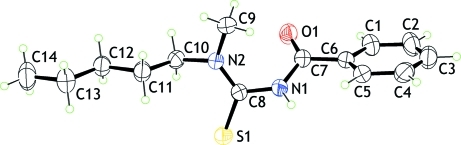
The molecular structure of the first independent molecule of (I) showing displacement ellipsoids at the 35% probability level.

**Fig. 2.**
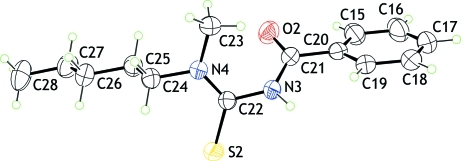
The molecular structure of the second independent molecule of (I) showing displacement ellipsoids at the 35% probability level.

**Fig. 3.**
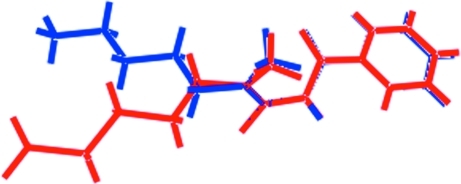
Overlay diagram showing the superimposition of the S1-containing molecule (red) with the S2-containing molecule (blue).

**Fig. 4.**
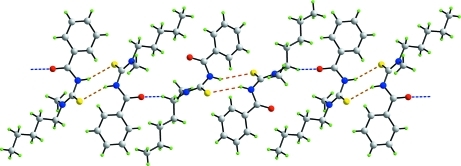
Supramolecular chain in (I) mediated by N—H···S hydrogen bonding and C—H···O contacts shown as orange and blue dashed lines, respectively.

## supplementary crystallographic information

### Comment

Thiourea derivatives exhibit remarkable coordination versatility towards metal cations (Pisiewicz *et al.*, 2010). In continuation of structural studies of thiourea derivatives (Gunasekaran *et al.*, 2010*a*; Gunasekaran *et al.*, 2010*b*; Gunasekaran *et al.*, 2010*c*), which have applications in the field of pharmaceuticals (Venkatachalam *et al.*, 2004; Bruce *et al.*, 2007) and catalysis (Gunasekaran *et al.*, 2010; Gunasekaran *et al.*, 2011), the crystal structure of the title compound, (I), was investigated.

Two independent molecules, Figs 1 and 2, comprise the asymmetric unit of (I). While similar, the molecules differ in the relative orientations of the pentyl groups, Fig. 3, as quantified in the values of the C10—C11—C12—C13 and C24—C25—C26—C27 torsion angles of -176.7 (3) and 176.4 (3) °, respectively, which indicate opposite orientations with respect to the remaining part of the respective molecules. While 50:50 disorder was found in the orientation of the phenyl ring in the second independent molecule, it is noted that the disordered rings are co-planar; dihedral angle = 6.9 (3) °. Significant twists are evident in each molecule with the dihedral angle formed between the thiourea and amide residues being 53.47 (17) ° [for S1,N1,N2,C7/O1,N1,C7,C7] and 55.81 (17) ° [for S2,N3,N4,C22/O2,N3,C21,C22]. Similarly, the terminal benzene ring is twisted out of the least-squares plane through the amide forming a C1—C6—C7—O1 torsion angle of -27.5 (4) °. For the second molecule, with disorder in the benzene ring, the C19—C20—C21—O2 and C19'—C20'—C21—O2 torsion angles are 154.8 (5) and 141.8 (6) °, respectively.

In the crystal packing, each independent molecule self-associates *via* a centrosymmetric eight-membered {···HNC═S}_2_ synthon. The carbonyl-O2 atom forms an intermolecular C—H···O interaction that serves to link the centrosymmetric dimers into a linear supramolecular chain along [110], Fig. 4; the carbonyl-O1 atom is engaged in an intramolecular C—H···O contact.

### Experimental

A solution of benzoyl chloride (0.7029 g, 5 mmol) in acetone (50 ml) was added drop-wise to a suspension of potassium thiocyanate (0.4859 g, 5 mmol) in anhydrous acetone (50 ml). The reaction mixture was heated under reflux for 45 min. and then cooled to room temperature. A solution of *n*-methylpentylamine (0.5060 g, 5 mmol) in acetone (30 ml) was added and the resulting mixture was stirred for 2 h. Hydrochloric acid (0.1 N, 300 ml) was added and the resulting white solid was filtered, washed with water and dried *in vacuo*. Crystals were grown at room temperature from its acetone solution. *M*.pt. 332–334 K; Yield 70%. FT—IR (KBr) ν(N—H) 3172, ν(C═O) 1685, ν(C═S) 1245 cm^-1^.

### Refinement

The H-atoms were placed in calculated positions (N—H = 0.88; C—H 0.93 to 0.97 Å) and were included in the refinement in the riding model approximation, with *U*_iso_(H) set to 1.2 to 1.5*U*_equiv_(N, C). The phenyl ring of the second of the independent molecules is disordered over two postions. The occupancy could not be refined, so the disorder was assumed to be a 1:1 type. The rings were refined as rigid hexagons of 1.39 Å sides. The anisotropic displacement factors of the primed atoms were set to those of the unprimed ones, and were restrained to be nearly isotropic. The C_carbonyl_–C_phenyl_ distances were restrained to within 0.01 Å of each other.

### Crystal data

**Table:**

C_14_H_20_N_2_OS	*Z* = 4
*M**_r_* = 264.38	*F*(000) = 568
Triclinic, *P*1	*D*_x_ = 1.183 Mg m^−^^3^
Hall symbol: -P 1	Mo *K*α radiation, λ = 0.71073 Å
*a* = 9.0992 (6) Å	Cell parameters from 2904 reflections
*b* = 10.5297 (6) Å	θ = 2.3–29.3°
*c* = 16.4038 (8) Å	µ = 0.21 mm^−^^1^
α = 75.784 (5)°	*T* = 295 K
β = 77.831 (5)°	Block, colourless
γ = 82.877 (5)°	0.25 × 0.20 × 0.15 mm
*V* = 1484.98 (15) Å^3^	

### Data collection

**Table:**

Agilent Supernova Dual diffractometer with an Atlas detector	6585 independent reflections
Radiation source: SuperNova (Mo) X-ray Source	3555 reflections with *I* > 2σ(*I*)
Mirror	*R*_int_ = 0.034
Detector resolution: 10.4041 pixels mm^-1^	θ_max_ = 27.5°, θ_min_ = 2.3°
ω scans	*h* = −11→11
Absorption correction: multi-scan (*CrysAlis PRO*; Agilent, 2010)	*k* = −13→13
*T*_min_ = 0.853, *T*_max_ = 1.000	*l* = −21→17
11884 measured reflections	

### Refinement

**Table:**

Refinement on *F*^2^	Primary atom site location: structure-invariant direct methods
Least-squares matrix: full	Secondary atom site location: difference Fourier map
*R*[*F*^2^ > 2σ(*F*^2^)] = 0.062	Hydrogen site location: inferred from neighbouring sites
*wR*(*F*^2^) = 0.184	H-atom parameters constrained
*S* = 1.03	*w* = 1/[σ^2^(*F*_o_^2^) + (0.071*P*)^2^ + 0.1338*P*] where *P* = (*F*_o_^2^ + 2*F*_c_^2^)/3
6585 reflections	(Δ/σ)_max_ = 0.001
321 parameters	Δρ_max_ = 0.33 e Å^−^^3^
37 restraints	Δρ_min_ = −0.27 e Å^−^^3^

### Special details

**Table:**

Geometry. All e.s.d.'s (except the e.s.d. in the dihedral angle between two l.s. planes) are estimated using the full covariance matrix. The cell e.s.d.'s are taken into account individually in the estimation of e.s.d.'s in distances, angles and torsion angles; correlations between e.s.d.'s in cell parameters are only used when they are defined by crystal symmetry. An approximate (isotropic) treatment of cell e.s.d.'s is used for estimating e.s.d.'s involving l.s. planes.
Refinement. Refinement of *F*^2^ against ALL reflections. The weighted *R*-factor *wR* and goodness of fit *S* are based on *F*^2^, conventional *R*-factors *R* are based on *F*, with *F* set to zero for negative *F*^2^. The threshold expression of *F*^2^ > σ(*F*^2^) is used only for calculating *R*-factors(gt) *etc*. and is not relevant to the choice of reflections for refinement. *R*-factors based on *F*^2^ are statistically about twice as large as those based on *F*, and *R*- factors based on ALL data will be even larger.

### Fractional atomic coordinates and isotropic or equivalent isotropic displacement parameters (Å^2^)

**Table:**

	*x*	*y*	*z*	*U*_iso_*/*U*_eq_	Occ. (<1)
S1	0.61942 (10)	0.45168 (7)	0.37694 (5)	0.0643 (3)	
S2	0.53313 (10)	1.00478 (8)	0.86780 (5)	0.0709 (3)	
O1	0.9407 (2)	0.6383 (2)	0.43989 (13)	0.0707 (6)	
O2	0.9060 (2)	1.1826 (2)	0.88476 (13)	0.0753 (6)	
N1	0.6894 (3)	0.6324 (2)	0.44660 (14)	0.0531 (6)	
H1	0.5983	0.6498	0.4746	0.064*	
N2	0.7833 (3)	0.6553 (2)	0.30138 (13)	0.0522 (6)	
N3	0.6572 (3)	1.1566 (2)	0.93652 (13)	0.0548 (6)	
H3	0.5849	1.1562	0.9816	0.066*	
N4	0.6452 (2)	1.2346 (2)	0.79196 (13)	0.0504 (5)	
C1	0.8832 (4)	0.6575 (3)	0.61411 (19)	0.0687 (9)	
H1A	0.9727	0.6093	0.5978	0.082*	
C2	0.8564 (5)	0.6965 (3)	0.6908 (2)	0.0818 (10)	
H2	0.9271	0.6727	0.7265	0.098*	
C3	0.7272 (5)	0.7696 (3)	0.7145 (2)	0.0810 (11)	
H3A	0.7106	0.7961	0.7660	0.097*	
C4	0.6215 (4)	0.8042 (3)	0.6629 (2)	0.0735 (9)	
H4A	0.5337	0.8548	0.6789	0.088*	
C5	0.6465 (4)	0.7633 (3)	0.58688 (18)	0.0616 (8)	
H5	0.5743	0.7854	0.5522	0.074*	
C6	0.7779 (3)	0.6897 (3)	0.56196 (17)	0.0531 (7)	
C7	0.8127 (3)	0.6516 (3)	0.47817 (17)	0.0537 (7)	
C8	0.7042 (3)	0.5864 (2)	0.37203 (16)	0.0492 (6)	
C9	0.8225 (4)	0.7902 (3)	0.29168 (19)	0.0658 (8)	
H9A	0.7595	0.8278	0.3359	0.099*	
H9B	0.8073	0.8420	0.2367	0.099*	
H9C	0.9263	0.7888	0.2960	0.099*	
C10	0.8343 (3)	0.6011 (3)	0.22507 (17)	0.0571 (7)	
H10A	0.8415	0.5060	0.2427	0.069*	
H10B	0.9346	0.6280	0.1982	0.069*	
C11	0.7337 (3)	0.6426 (3)	0.16005 (17)	0.0624 (8)	
H11A	0.7196	0.7377	0.1454	0.075*	
H11B	0.6357	0.6088	0.1848	0.075*	
C12	0.7986 (4)	0.5931 (3)	0.07880 (18)	0.0645 (8)	
H12A	0.8938	0.6311	0.0524	0.077*	
H12B	0.8189	0.4985	0.0942	0.077*	
C13	0.6965 (4)	0.6257 (4)	0.0148 (2)	0.0817 (10)	
H13A	0.6789	0.7204	−0.0020	0.098*	
H13B	0.6002	0.5900	0.0417	0.098*	
C14	0.7584 (5)	0.5731 (4)	−0.0640 (2)	0.1030 (14)	
H14A	0.6881	0.5970	−0.1024	0.154*	
H14B	0.7741	0.4792	−0.0480	0.154*	
H14C	0.8526	0.6097	−0.0918	0.154*	
C15	0.9809 (9)	1.1289 (13)	1.0465 (5)	0.0735 (18)	0.50
H15	1.0559	1.1012	1.0051	0.088*	0.50
C16	1.0117 (6)	1.1274 (10)	1.1263 (5)	0.089 (2)	0.50
H16	1.1074	1.0987	1.1382	0.107*	0.50
C17	0.8995 (7)	1.1687 (8)	1.1882 (3)	0.087 (3)	0.50
H17	0.9202	1.1677	1.2415	0.104*	0.50
C18	0.7565 (6)	1.2116 (8)	1.1703 (4)	0.082 (2)	0.50
H18	0.6815	1.2393	1.2117	0.099*	0.50
C19	0.7257 (8)	1.2131 (11)	1.0906 (5)	0.0648 (19)	0.50
H19	0.6300	1.2418	1.0787	0.078*	0.50
C20	0.8379 (10)	1.1718 (14)	1.0287 (4)	0.0552 (16)	0.50
C15'	0.9290 (9)	1.1414 (12)	1.0646 (5)	0.0735 (18)	0.50
H15'	1.0083	1.1017	1.0314	0.088*	0.50
C16'	0.9421 (7)	1.1526 (10)	1.1454 (5)	0.089 (2)	0.50
H16'	1.0301	1.1204	1.1662	0.107*	0.50
C17'	0.8235 (8)	1.2120 (8)	1.1952 (3)	0.087 (3)	0.50
H17'	0.8323	1.2195	1.2492	0.104*	0.50
C18'	0.6918 (7)	1.2602 (8)	1.1641 (4)	0.082 (2)	0.50
H18'	0.6125	1.2999	1.1973	0.099*	0.50
C19'	0.6788 (8)	1.2490 (11)	1.0833 (5)	0.0648 (19)	0.50
H19'	0.5907	1.2812	1.0625	0.078*	0.50
C20'	0.7973 (11)	1.1896 (13)	1.0335 (4)	0.0552 (16)	0.50
C21	0.8010 (4)	1.1734 (3)	0.94459 (18)	0.0573 (7)	
C22	0.6179 (3)	1.1403 (3)	0.86181 (16)	0.0498 (6)	
C23	0.7007 (4)	1.3610 (3)	0.78851 (19)	0.0654 (8)	
H23A	0.6846	1.3758	0.8453	0.098*	
H23B	0.6474	1.4302	0.7534	0.098*	
H23C	0.8065	1.3599	0.7645	0.098*	
C24	0.6123 (3)	1.2216 (3)	0.71090 (17)	0.0564 (7)	
H24A	0.5828	1.3080	0.6787	0.068*	
H24B	0.5274	1.1682	0.7230	0.068*	
C25	0.7425 (3)	1.1613 (3)	0.65679 (17)	0.0612 (8)	
H25A	0.7716	1.0741	0.6880	0.073*	
H25B	0.8280	1.2142	0.6442	0.073*	
C26	0.7007 (4)	1.1520 (3)	0.57313 (18)	0.0655 (8)	
H26A	0.6110	1.1041	0.5864	0.079*	
H26B	0.6764	1.2399	0.5412	0.079*	
C27	0.8234 (4)	1.0852 (3)	0.51769 (19)	0.0768 (9)	
H27A	0.8489	0.9977	0.5499	0.092*	
H27B	0.9126	1.1339	0.5036	0.092*	
C28	0.7804 (5)	1.0744 (4)	0.4357 (2)	0.0996 (13)	
H28A	0.8630	1.0315	0.4031	0.149*	
H28B	0.7569	1.1607	0.4029	0.149*	
H28C	0.6938	1.0242	0.4491	0.149*	

### Atomic displacement parameters (Å^2^)

**Table:**

	*U*^11^	*U*^22^	*U*^33^	*U*^12^	*U*^13^	*U*^23^
S1	0.0813 (6)	0.0575 (4)	0.0548 (5)	−0.0222 (4)	−0.0078 (4)	−0.0099 (3)
S2	0.0970 (7)	0.0662 (5)	0.0537 (5)	−0.0357 (5)	−0.0040 (4)	−0.0159 (4)
O1	0.0542 (14)	0.1030 (16)	0.0591 (13)	−0.0065 (12)	−0.0091 (11)	−0.0271 (12)
O2	0.0600 (14)	0.1055 (17)	0.0560 (13)	−0.0133 (12)	−0.0061 (11)	−0.0105 (12)
N1	0.0473 (14)	0.0685 (14)	0.0470 (13)	−0.0106 (11)	−0.0049 (10)	−0.0201 (11)
N2	0.0558 (14)	0.0595 (13)	0.0433 (12)	−0.0143 (11)	−0.0056 (10)	−0.0136 (11)
N3	0.0578 (15)	0.0684 (14)	0.0399 (12)	−0.0169 (12)	−0.0009 (10)	−0.0165 (11)
N4	0.0537 (14)	0.0545 (13)	0.0438 (12)	−0.0105 (11)	−0.0081 (10)	−0.0103 (10)
C1	0.079 (2)	0.0736 (19)	0.0589 (19)	−0.0030 (17)	−0.0228 (17)	−0.0192 (16)
C2	0.115 (3)	0.081 (2)	0.059 (2)	−0.011 (2)	−0.036 (2)	−0.0166 (18)
C3	0.120 (3)	0.072 (2)	0.0526 (19)	−0.020 (2)	−0.006 (2)	−0.0202 (17)
C4	0.082 (2)	0.068 (2)	0.067 (2)	−0.0166 (18)	0.0057 (19)	−0.0202 (17)
C5	0.067 (2)	0.0676 (18)	0.0528 (17)	−0.0205 (16)	−0.0036 (15)	−0.0167 (14)
C6	0.0601 (19)	0.0543 (15)	0.0464 (15)	−0.0152 (14)	−0.0063 (14)	−0.0120 (13)
C7	0.0560 (19)	0.0601 (17)	0.0463 (15)	−0.0085 (14)	−0.0105 (14)	−0.0116 (13)
C8	0.0487 (16)	0.0552 (15)	0.0455 (15)	−0.0074 (13)	−0.0100 (12)	−0.0115 (12)
C9	0.074 (2)	0.0660 (18)	0.0574 (18)	−0.0282 (16)	−0.0030 (15)	−0.0107 (15)
C10	0.0505 (17)	0.0713 (18)	0.0497 (16)	−0.0064 (14)	−0.0026 (13)	−0.0190 (14)
C11	0.062 (2)	0.0771 (19)	0.0505 (17)	−0.0022 (16)	−0.0114 (14)	−0.0184 (15)
C12	0.075 (2)	0.0686 (18)	0.0514 (17)	−0.0088 (16)	−0.0110 (15)	−0.0160 (14)
C13	0.095 (3)	0.094 (2)	0.062 (2)	0.000 (2)	−0.0252 (19)	−0.0226 (18)
C14	0.157 (4)	0.100 (3)	0.062 (2)	−0.013 (3)	−0.034 (2)	−0.025 (2)
C15	0.085 (6)	0.069 (3)	0.074 (3)	−0.011 (4)	−0.028 (4)	−0.016 (3)
C16	0.110 (6)	0.086 (4)	0.084 (4)	−0.017 (4)	−0.049 (5)	−0.011 (3)
C17	0.108 (7)	0.101 (5)	0.061 (3)	−0.042 (5)	−0.030 (4)	−0.008 (3)
C18	0.096 (6)	0.097 (6)	0.060 (3)	−0.043 (4)	0.000 (3)	−0.023 (3)
C19	0.066 (5)	0.077 (5)	0.053 (2)	−0.026 (4)	−0.007 (2)	−0.012 (3)
C20	0.059 (5)	0.055 (3)	0.0513 (19)	−0.017 (3)	−0.014 (2)	−0.0020 (16)
C15'	0.085 (6)	0.069 (3)	0.074 (3)	−0.011 (4)	−0.028 (4)	−0.016 (3)
C16'	0.110 (6)	0.086 (4)	0.084 (4)	−0.017 (4)	−0.049 (5)	−0.011 (3)
C17'	0.108 (7)	0.101 (5)	0.061 (3)	−0.042 (5)	−0.030 (4)	−0.008 (3)
C18'	0.096 (6)	0.097 (6)	0.060 (3)	−0.043 (4)	0.000 (3)	−0.023 (3)
C19'	0.066 (5)	0.077 (5)	0.053 (2)	−0.026 (4)	−0.007 (2)	−0.012 (3)
C20'	0.059 (5)	0.055 (3)	0.0513 (19)	−0.017 (3)	−0.014 (2)	−0.0020 (16)
C21	0.069 (2)	0.0562 (16)	0.0464 (16)	−0.0165 (15)	−0.0122 (15)	−0.0049 (13)
C22	0.0520 (17)	0.0552 (15)	0.0444 (15)	−0.0106 (13)	−0.0048 (12)	−0.0158 (13)
C23	0.077 (2)	0.0569 (16)	0.0643 (19)	−0.0220 (15)	−0.0176 (16)	−0.0047 (15)
C24	0.0570 (18)	0.0629 (17)	0.0504 (16)	−0.0062 (14)	−0.0157 (14)	−0.0096 (13)
C25	0.0575 (19)	0.0749 (19)	0.0518 (17)	−0.0068 (15)	−0.0125 (14)	−0.0126 (15)
C26	0.072 (2)	0.0721 (19)	0.0501 (17)	−0.0020 (16)	−0.0134 (15)	−0.0093 (15)
C27	0.083 (2)	0.088 (2)	0.0565 (19)	−0.0053 (19)	−0.0004 (17)	−0.0212 (17)
C28	0.142 (4)	0.101 (3)	0.053 (2)	0.013 (3)	−0.015 (2)	−0.0249 (19)

### Geometric parameters (Å, °)

**Table:**

S1—C8	1.674 (3)	C14—H14B	0.9600
S2—C22	1.677 (3)	C14—H14C	0.9600
O1—C7	1.210 (3)	C15—C16	1.3900
O2—C21	1.211 (3)	C15—C20	1.3900
N1—C7	1.387 (3)	C15—H15	0.9300
N1—C8	1.398 (3)	C16—C17	1.3900
N1—H1	0.8800	C16—H16	0.9300
N2—C8	1.324 (3)	C17—C18	1.3900
N2—C10	1.466 (3)	C17—H17	0.9300
N2—C9	1.470 (3)	C18—C19	1.3900
N3—C21	1.379 (4)	C18—H18	0.9300
N3—C22	1.401 (3)	C19—C20	1.3900
N3—H3	0.8800	C19—H19	0.9300
N4—C22	1.320 (3)	C20—C21	1.483 (5)
N4—C24	1.464 (3)	C15'—C16'	1.3900
N4—C23	1.466 (3)	C15'—C20'	1.3900
C1—C6	1.374 (4)	C15'—H15'	0.9300
C1—C2	1.383 (4)	C16'—C17'	1.3900
C1—H1A	0.9300	C16'—H16'	0.9300
C2—C3	1.362 (5)	C17'—C18'	1.3900
C2—H2	0.9300	C17'—H17'	0.9300
C3—C4	1.371 (5)	C18'—C19'	1.3900
C3—H3A	0.9300	C18'—H18'	0.9300
C4—C5	1.383 (4)	C19'—C20'	1.3900
C4—H4A	0.9300	C19'—H19'	0.9300
C5—C6	1.385 (4)	C20'—C21	1.503 (5)
C5—H5	0.9300	C23—H23A	0.9600
C6—C7	1.485 (4)	C23—H23B	0.9600
C9—H9A	0.9600	C23—H23C	0.9600
C9—H9B	0.9600	C24—C25	1.498 (4)
C9—H9C	0.9600	C24—H24A	0.9700
C10—C11	1.500 (4)	C24—H24B	0.9700
C10—H10A	0.9700	C25—C26	1.528 (4)
C10—H10B	0.9700	C25—H25A	0.9700
C11—C12	1.526 (4)	C25—H25B	0.9700
C11—H11A	0.9700	C26—C27	1.503 (4)
C11—H11B	0.9700	C26—H26A	0.9700
C12—C13	1.495 (4)	C26—H26B	0.9700
C12—H12A	0.9700	C27—C28	1.511 (5)
C12—H12B	0.9700	C27—H27A	0.9700
C13—C14	1.503 (4)	C27—H27B	0.9700
C13—H13A	0.9700	C28—H28A	0.9600
C13—H13B	0.9700	C28—H28B	0.9600
C14—H14A	0.9600	C28—H28C	0.9600
			
C7—N1—C8	122.5 (2)	C15—C16—H16	120.0
C7—N1—H1	118.8	C16—C17—C18	120.0
C8—N1—H1	118.8	C16—C17—H17	120.0
C8—N2—C10	121.0 (2)	C18—C17—H17	120.0
C8—N2—C9	123.9 (2)	C17—C18—C19	120.0
C10—N2—C9	115.1 (2)	C17—C18—H18	120.0
C21—N3—C22	124.8 (2)	C19—C18—H18	120.0
C21—N3—H3	117.6	C20—C19—C18	120.0
C22—N3—H3	117.6	C20—C19—H19	120.0
C22—N4—C24	120.8 (2)	C18—C19—H19	120.0
C22—N4—C23	124.7 (2)	C19—C20—C15	120.0
C24—N4—C23	114.4 (2)	C19—C20—C21	118.7 (5)
C6—C1—C2	120.2 (3)	C15—C20—C21	121.3 (5)
C6—C1—H1A	119.9	C16'—C15'—C20'	120.0
C2—C1—H1A	119.9	C16'—C15'—H15'	120.0
C3—C2—C1	120.4 (3)	C20'—C15'—H15'	120.0
C3—C2—H2	119.8	C15'—C16'—C17'	120.0
C1—C2—H2	119.8	C15'—C16'—H16'	120.0
C2—C3—C4	120.4 (3)	C17'—C16'—H16'	120.0
C2—C3—H3A	119.8	C18'—C17'—C16'	120.0
C4—C3—H3A	119.8	C18'—C17'—H17'	120.0
C3—C4—C5	119.4 (3)	C16'—C17'—H17'	120.0
C3—C4—H4A	120.3	C19'—C18'—C17'	120.0
C5—C4—H4A	120.3	C19'—C18'—H18'	120.0
C6—C5—C4	120.7 (3)	C17'—C18'—H18'	120.0
C6—C5—H5	119.7	C18'—C19'—C20'	120.0
C4—C5—H5	119.7	C18'—C19'—H19'	120.0
C1—C6—C5	119.0 (3)	C20'—C19'—H19'	120.0
C1—C6—C7	118.9 (3)	C19'—C20'—C15'	120.0
C5—C6—C7	122.0 (3)	C19'—C20'—C21	126.0 (6)
O1—C7—N1	122.1 (3)	C15'—C20'—C21	114.0 (6)
O1—C7—C6	122.1 (3)	O2—C21—N3	122.2 (3)
N1—C7—C6	115.8 (3)	O2—C21—C20	116.0 (5)
N2—C8—N1	116.8 (2)	N3—C21—C20	121.6 (5)
N2—C8—S1	124.5 (2)	O2—C21—C20'	128.5 (5)
N1—C8—S1	118.72 (19)	N3—C21—C20'	109.1 (5)
N2—C9—H9A	109.5	C20—C21—C20'	15.2 (6)
N2—C9—H9B	109.5	N4—C22—N3	117.9 (2)
H9A—C9—H9B	109.5	N4—C22—S2	124.2 (2)
N2—C9—H9C	109.5	N3—C22—S2	117.86 (19)
H9A—C9—H9C	109.5	N4—C23—H23A	109.5
H9B—C9—H9C	109.5	N4—C23—H23B	109.5
N2—C10—C11	114.3 (2)	H23A—C23—H23B	109.5
N2—C10—H10A	108.7	N4—C23—H23C	109.5
C11—C10—H10A	108.7	H23A—C23—H23C	109.5
N2—C10—H10B	108.7	H23B—C23—H23C	109.5
C11—C10—H10B	108.7	N4—C24—C25	113.6 (2)
H10A—C10—H10B	107.6	N4—C24—H24A	108.9
C10—C11—C12	112.1 (2)	C25—C24—H24A	108.9
C10—C11—H11A	109.2	N4—C24—H24B	108.9
C12—C11—H11A	109.2	C25—C24—H24B	108.9
C10—C11—H11B	109.2	H24A—C24—H24B	107.7
C12—C11—H11B	109.2	C24—C25—C26	110.7 (2)
H11A—C11—H11B	107.9	C24—C25—H25A	109.5
C13—C12—C11	113.5 (3)	C26—C25—H25A	109.5
C13—C12—H12A	108.9	C24—C25—H25B	109.5
C11—C12—H12A	108.9	C26—C25—H25B	109.5
C13—C12—H12B	108.9	H25A—C25—H25B	108.1
C11—C12—H12B	108.9	C27—C26—C25	113.6 (3)
H12A—C12—H12B	107.7	C27—C26—H26A	108.8
C12—C13—C14	113.4 (3)	C25—C26—H26A	108.8
C12—C13—H13A	108.9	C27—C26—H26B	108.8
C14—C13—H13A	108.9	C25—C26—H26B	108.8
C12—C13—H13B	108.9	H26A—C26—H26B	107.7
C14—C13—H13B	108.9	C26—C27—C28	113.3 (3)
H13A—C13—H13B	107.7	C26—C27—H27A	108.9
C13—C14—H14A	109.5	C28—C27—H27A	108.9
C13—C14—H14B	109.5	C26—C27—H27B	108.9
H14A—C14—H14B	109.5	C28—C27—H27B	108.9
C13—C14—H14C	109.5	H27A—C27—H27B	107.7
H14A—C14—H14C	109.5	C27—C28—H28A	109.5
H14B—C14—H14C	109.5	C27—C28—H28B	109.5
C16—C15—C20	120.0	H28A—C28—H28B	109.5
C16—C15—H15	120.0	C27—C28—H28C	109.5
C20—C15—H15	120.0	H28A—C28—H28C	109.5
C17—C16—C15	120.0	H28B—C28—H28C	109.5
C17—C16—H16	120.0		
			
C6—C1—C2—C3	−1.4 (5)	C15'—C16'—C17'—C18'	0.0
C1—C2—C3—C4	0.6 (5)	C16'—C17'—C18'—C19'	0.0
C2—C3—C4—C5	0.6 (5)	C17'—C18'—C19'—C20'	0.0
C3—C4—C5—C6	−1.0 (5)	C18'—C19'—C20'—C15'	0.0
C2—C1—C6—C5	1.0 (4)	C18'—C19'—C20'—C21	−178.4 (12)
C2—C1—C6—C7	177.3 (3)	C16'—C15'—C20'—C19'	0.0
C4—C5—C6—C1	0.2 (4)	C16'—C15'—C20'—C21	178.6 (10)
C4—C5—C6—C7	−175.9 (3)	C22—N3—C21—O2	4.2 (4)
C8—N1—C7—O1	5.1 (4)	C22—N3—C21—C20	−171.5 (6)
C8—N1—C7—C6	−174.9 (2)	C22—N3—C21—C20'	179.1 (6)
C1—C6—C7—O1	−27.5 (4)	C19—C20—C21—O2	154.8 (5)
C5—C6—C7—O1	148.7 (3)	C15—C20—C21—O2	−25.8 (9)
C1—C6—C7—N1	152.6 (3)	C19—C20—C21—N3	−29.3 (10)
C5—C6—C7—N1	−31.3 (4)	C15—C20—C21—N3	150.1 (5)
C10—N2—C8—N1	166.1 (2)	C19—C20—C21—C20'	7(4)
C9—N2—C8—N1	−15.9 (4)	C15—C20—C21—C20'	−174 (5)
C10—N2—C8—S1	−15.1 (4)	C19'—C20'—C21—O2	141.8 (6)
C9—N2—C8—S1	162.9 (2)	C15'—C20'—C21—O2	−36.7 (9)
C7—N1—C8—N2	−56.8 (3)	C19'—C20'—C21—N3	−32.8 (10)
C7—N1—C8—S1	124.4 (2)	C15'—C20'—C21—N3	148.7 (5)
C8—N2—C10—C11	96.6 (3)	C19'—C20'—C21—C20	179 (5)
C9—N2—C10—C11	−81.5 (3)	C15'—C20'—C21—C20	1(4)
N2—C10—C11—C12	175.1 (2)	C24—N4—C22—N3	177.6 (2)
C10—C11—C12—C13	176.4 (3)	C23—N4—C22—N3	−6.0 (4)
C11—C12—C13—C14	−178.2 (3)	C24—N4—C22—S2	−4.8 (4)
C20—C15—C16—C17	0.0	C23—N4—C22—S2	171.5 (2)
C15—C16—C17—C18	0.0	C21—N3—C22—N4	−59.2 (4)
C16—C17—C18—C19	0.0	C21—N3—C22—S2	123.0 (3)
C17—C18—C19—C20	0.0	C22—N4—C24—C25	−91.2 (3)
C18—C19—C20—C15	0.0	C23—N4—C24—C25	92.1 (3)
C18—C19—C20—C21	179.4 (11)	N4—C24—C25—C26	−179.5 (2)
C16—C15—C20—C19	0.0	C24—C25—C26—C27	−176.7 (3)
C16—C15—C20—C21	−179.4 (11)	C25—C26—C27—C28	179.1 (3)
C20'—C15'—C16'—C17'	0.0		

### Hydrogen-bond geometry (Å, °)

**Table:**

*D*—H···*A*	*D*—H	H···*A*	*D*···*A*	*D*—H···*A*
N1—H1···S1^i^	0.88	2.87	3.604 (2)	143
N3—H3···S2^ii^	0.88	2.72	3.449 (2)	141
C10—H10b···O2^iii^	0.97	2.55	3.397 (4)	146

Symmetry codes: (i) −*x*+1, −*y*+1, −*z*+1; (ii) −*x*+1, −*y*+2, −*z*+2; (iii) −*x*+2, −*y*+2, −*z*+1.
